# Supplementary figures and images for: Inferring repeat-protein energetics from evolutionary information
Source: PLoS Comput Biol. 2017 Jun 15;13(6):e1005584. doi: 10.1371/journal.pcbi.1005584 (PMC5491312; doi:10.1371/journal.pcbi.1005584)

# ANK

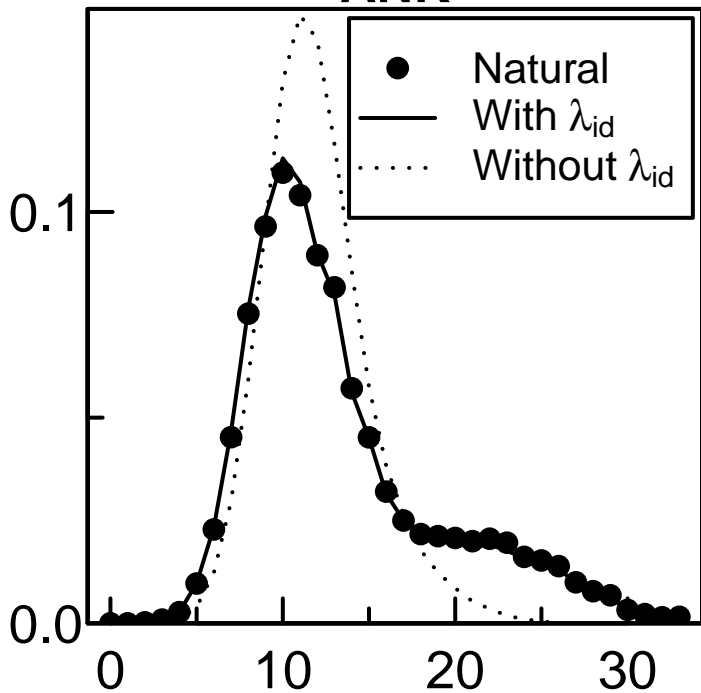

Supplement: S1 Fig — In black dots, the natural sequences’ distribution of %Id. (PDF) [file pcbi.1005584.s001.pdf]

ANK

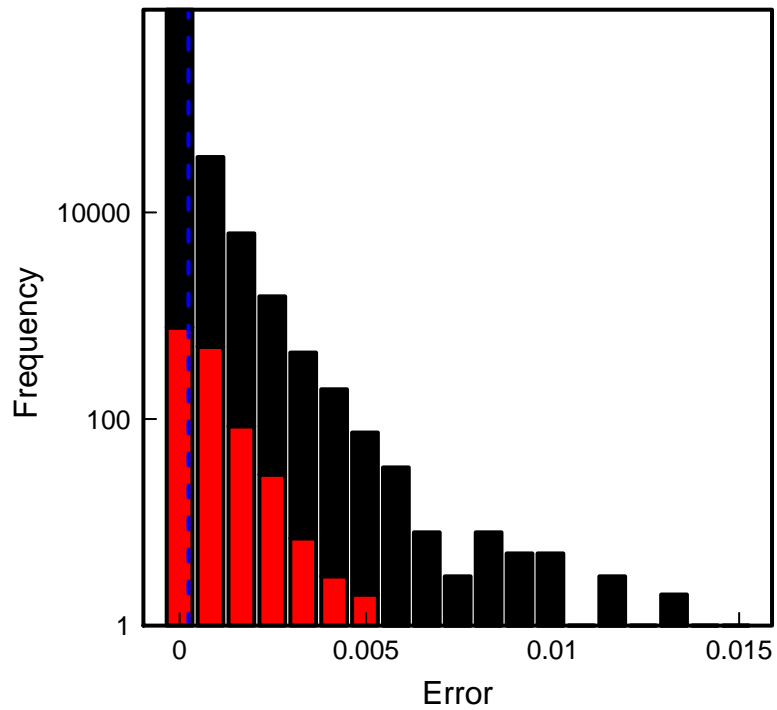

TPR

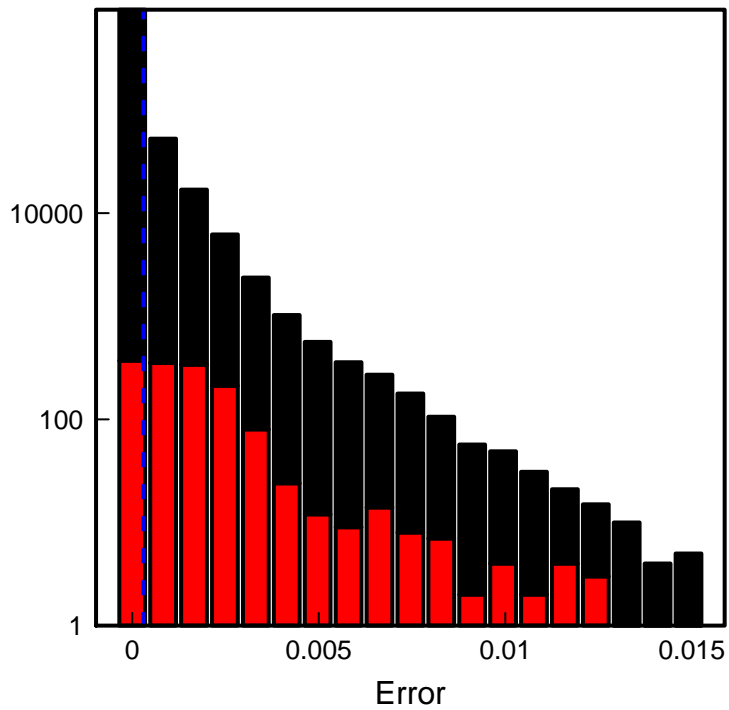

LRR

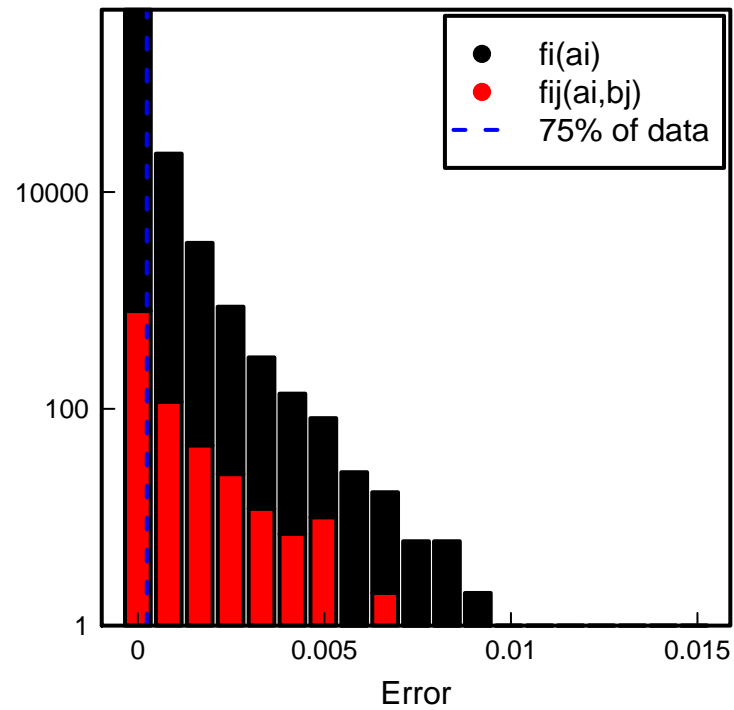

Supplement: S2 Fig — (PDF) [file pcbi.1005584.s002.pdf]

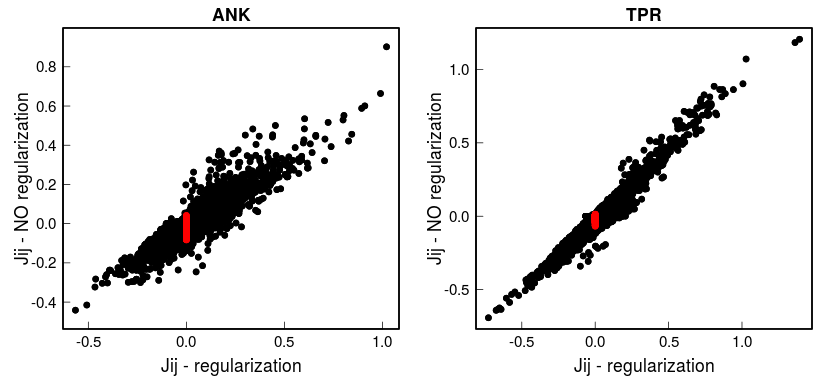

Supplement: S3 Fig — (PNG) [file pcbi.1005584.s003.png]

### ANK

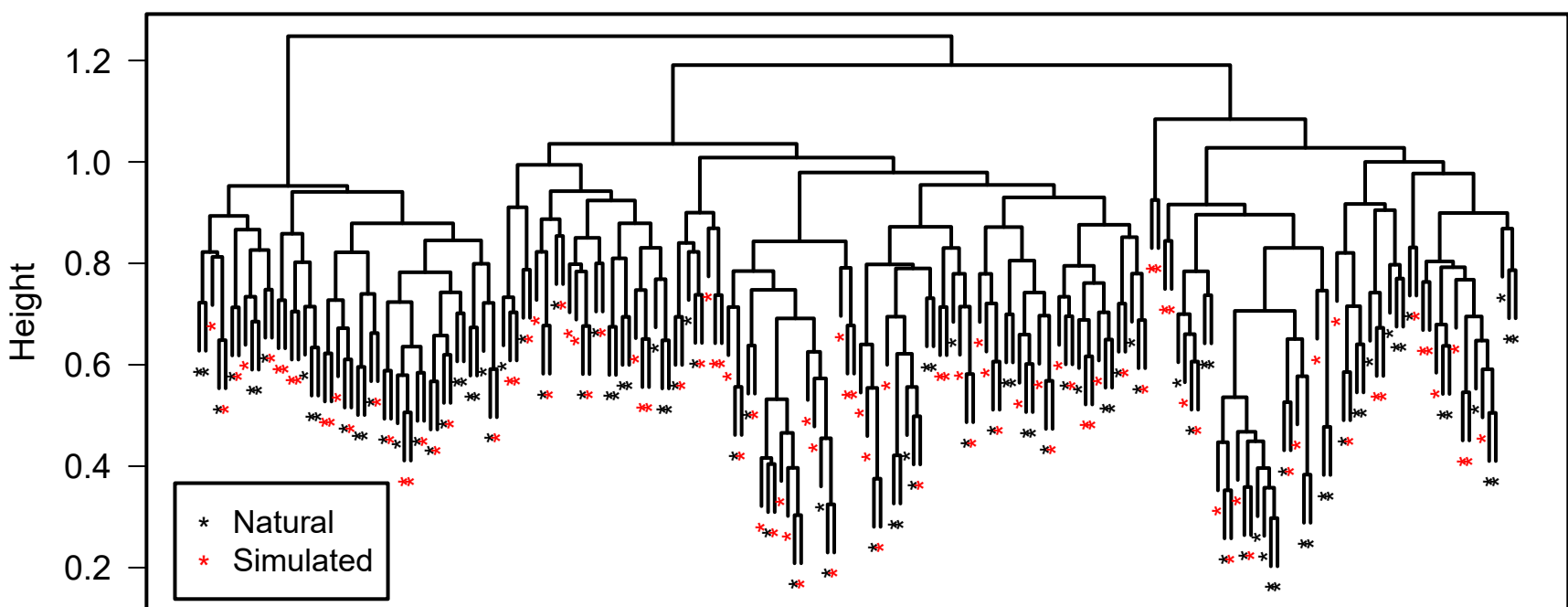

### TPR

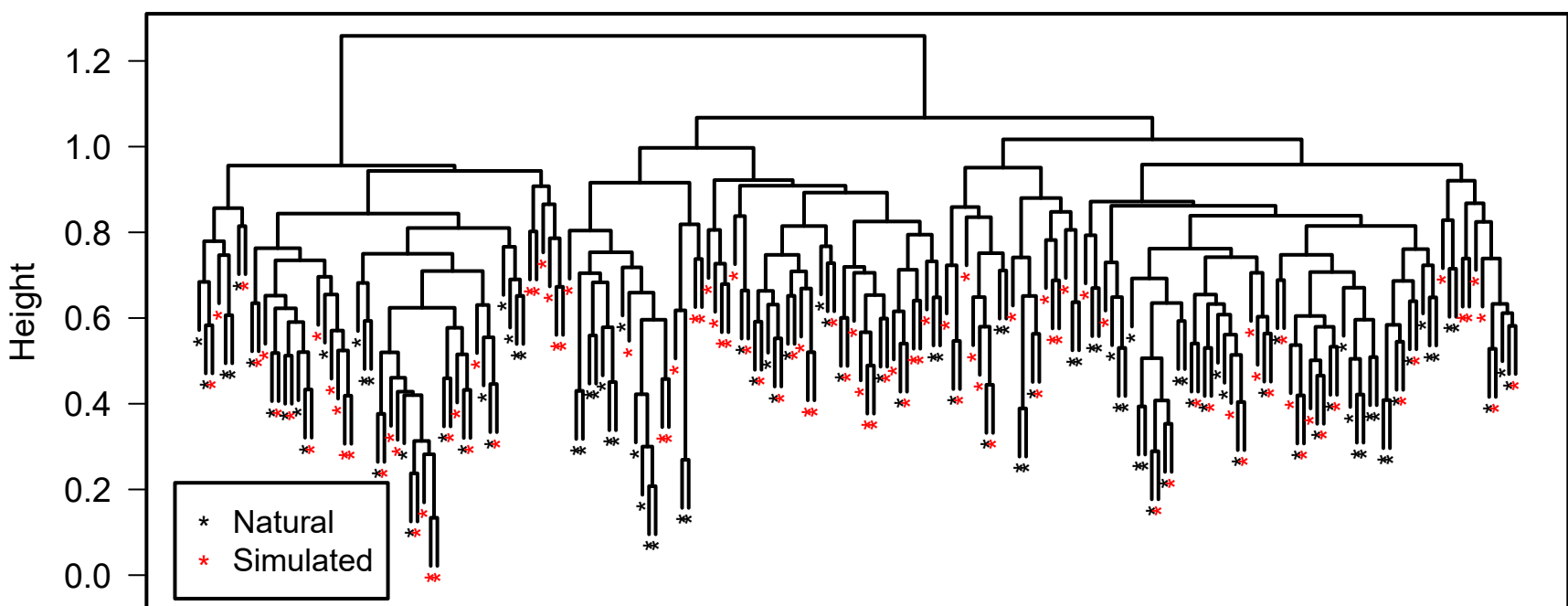

### LRR

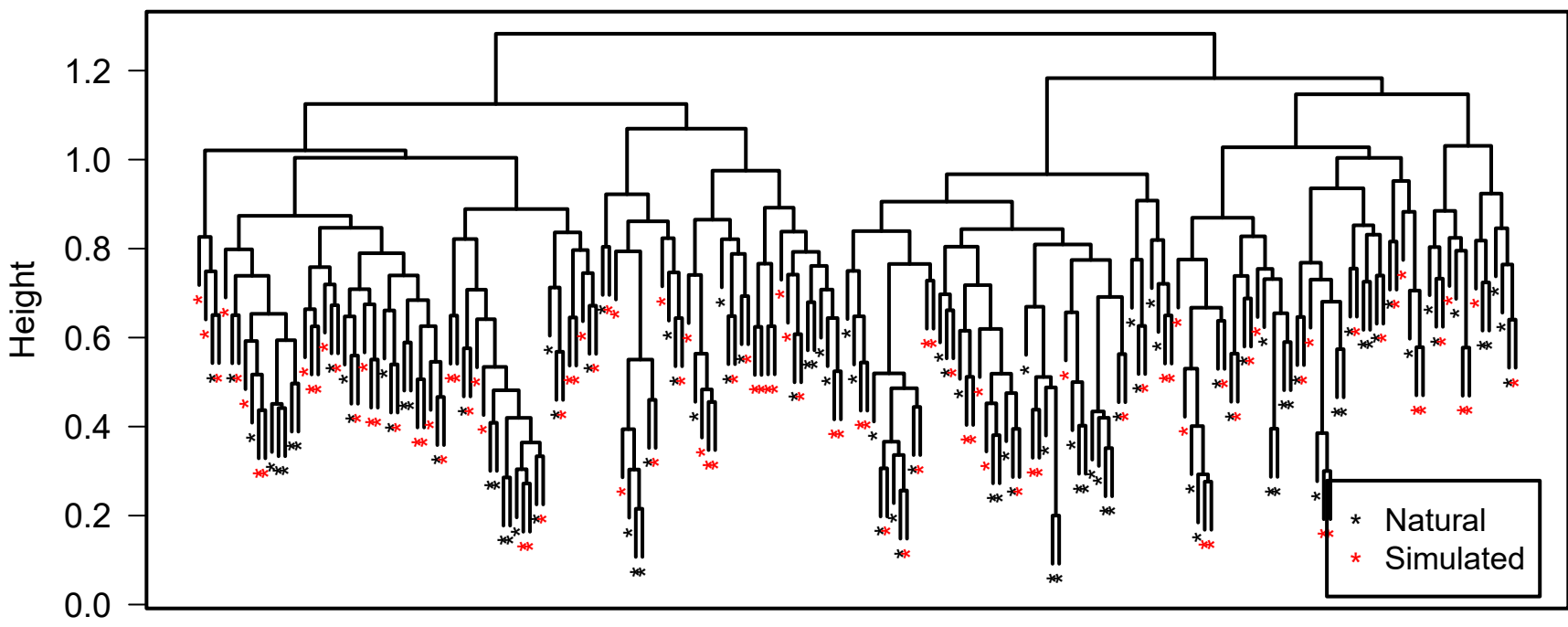

Supplement: S4 Fig — Natural and simulated sequences are indistinguishable from pairwise similarity. (PDF) [file pcbi.1005584.s004.pdf]

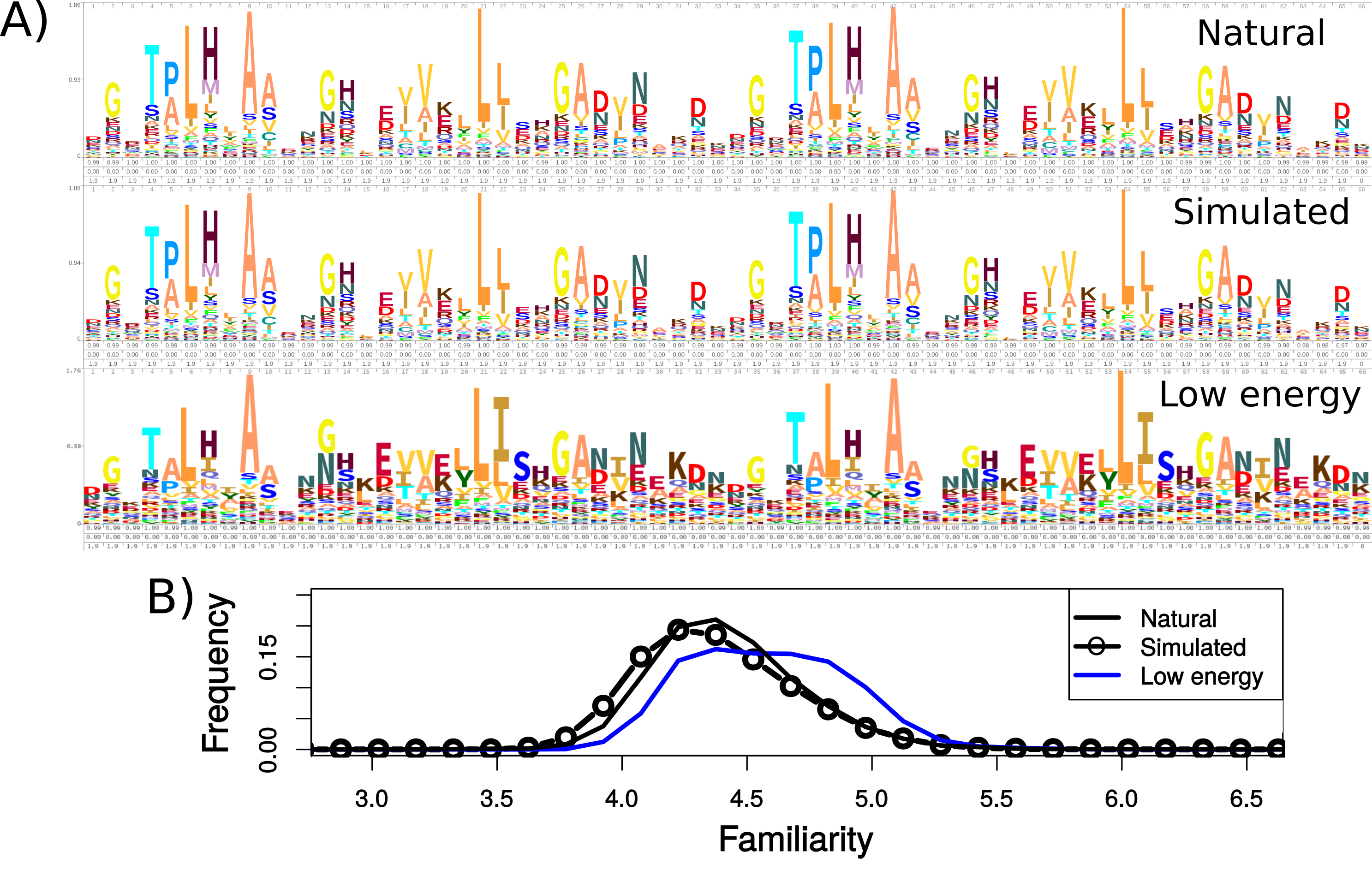

Supplement: S5 Fig — A) Logos for the MSA of natural pairs of ANK repeats (top), of simulated pairs of ANK repeats (center) and low energy pairs of simulated ANK repeats (bottom). B) Distribution of familiarity, as defined in Turjanski et al (2016) for the same sets of sequences. Simulated sequences reproduce the distribution of natural proteins and are indistinguishable. (PNG) [file pcbi.1005584.s005.png]

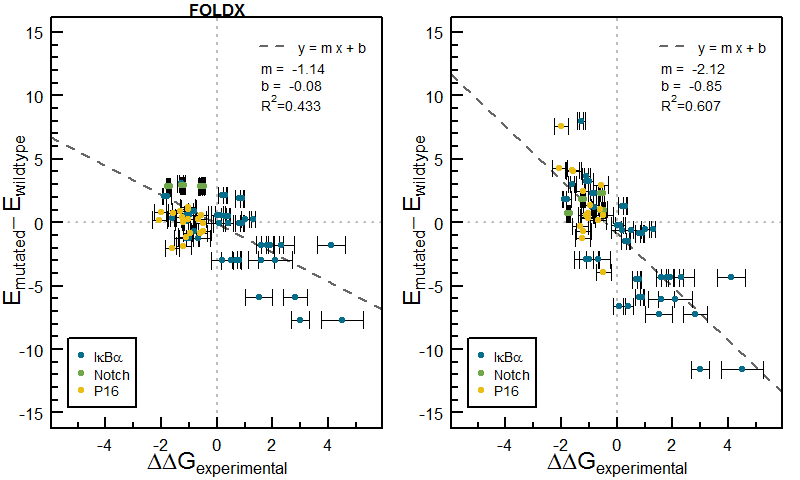

Supplement: S6 Fig — As FoldX requires a reference structure, some of the constructions tested in the protein Notch cannot be analyzed, so we excluded them from our predictions for a fair comparison. It can be seen that, overall, our model (right panel) is a better predictor than foldX (left panel) of stability changes upon mutation, and has the advantage of using just the sequence information. (PNG) [file pcbi.1005584.s006.png]

ANK

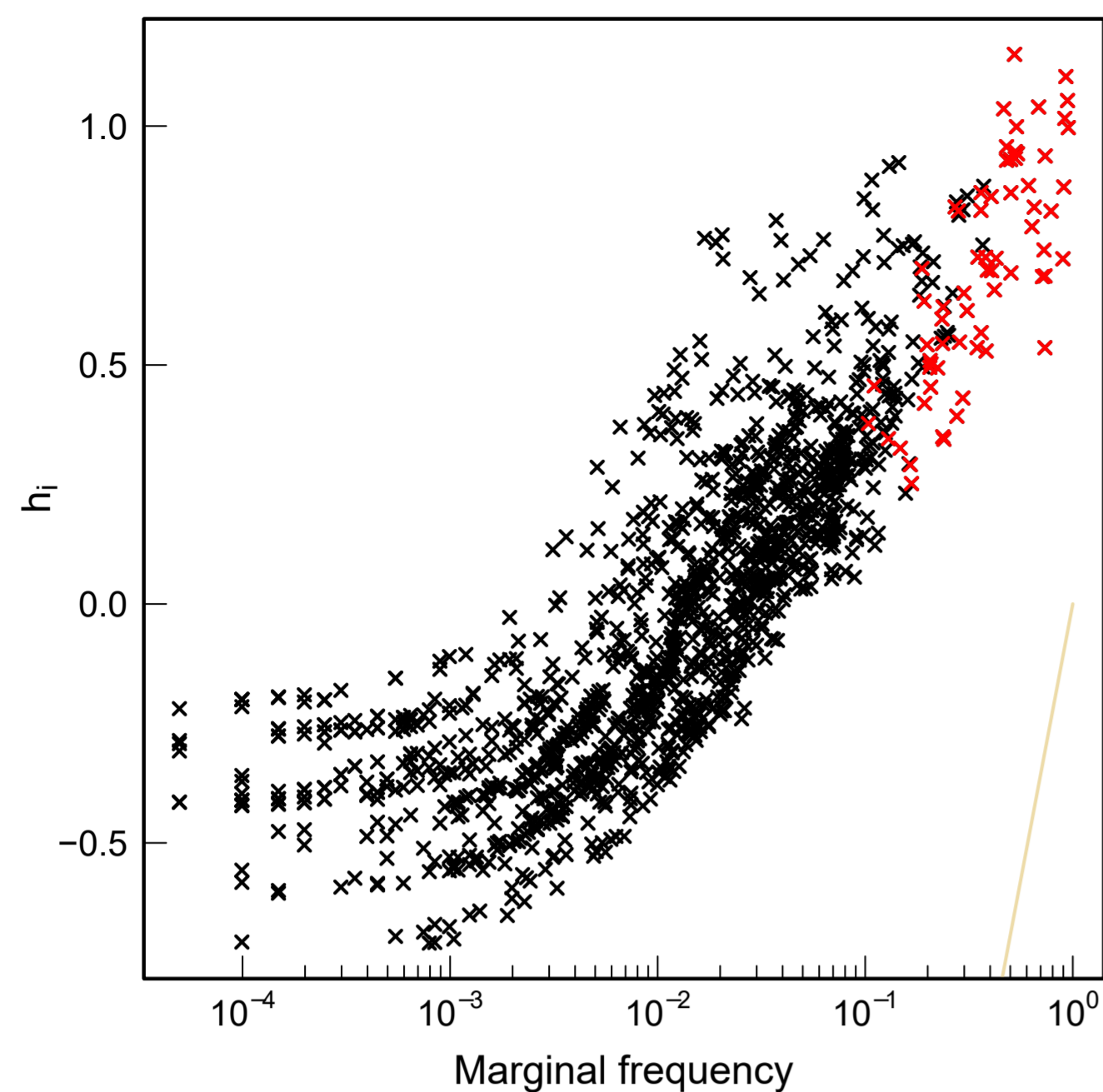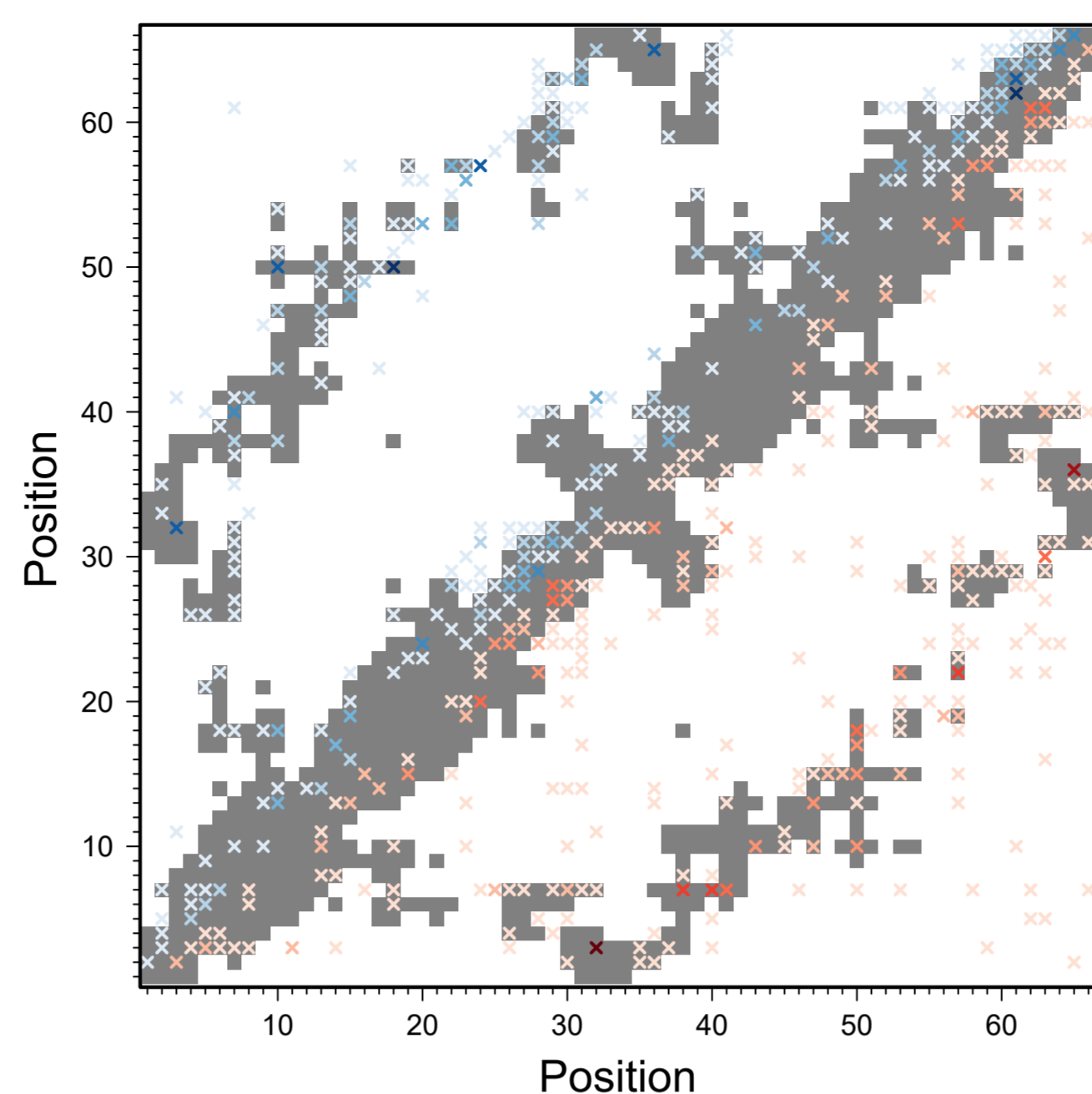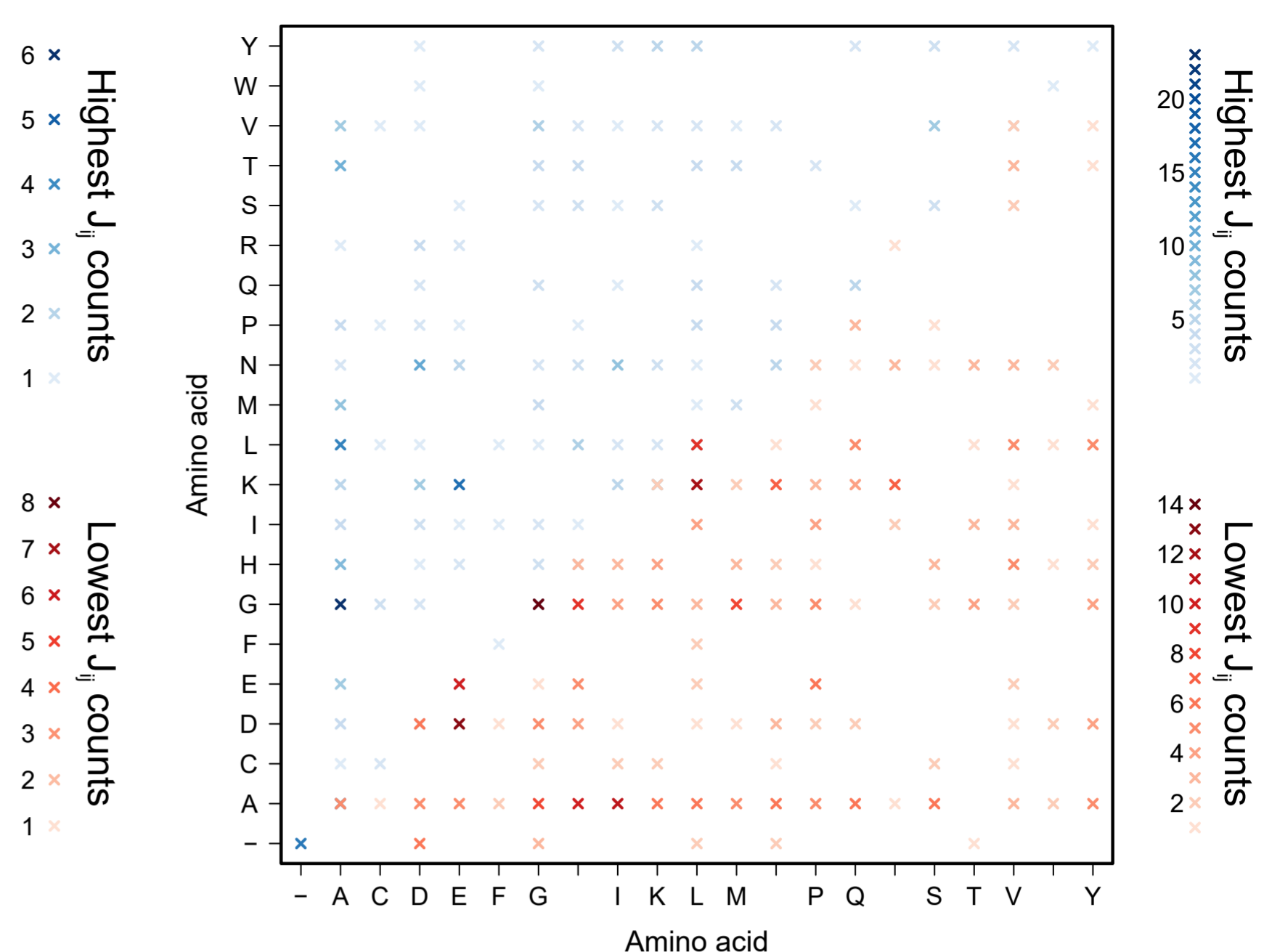

TPR

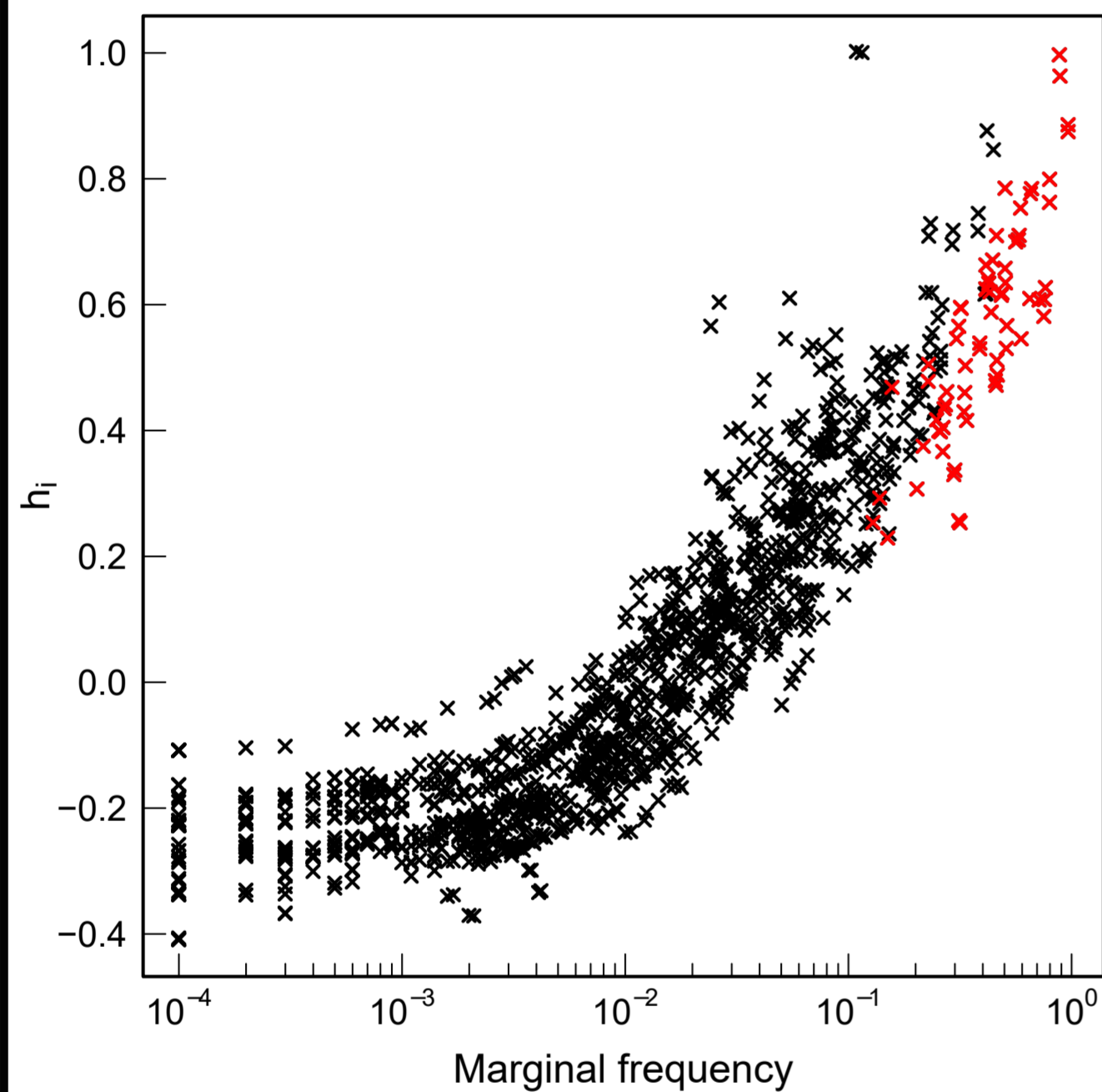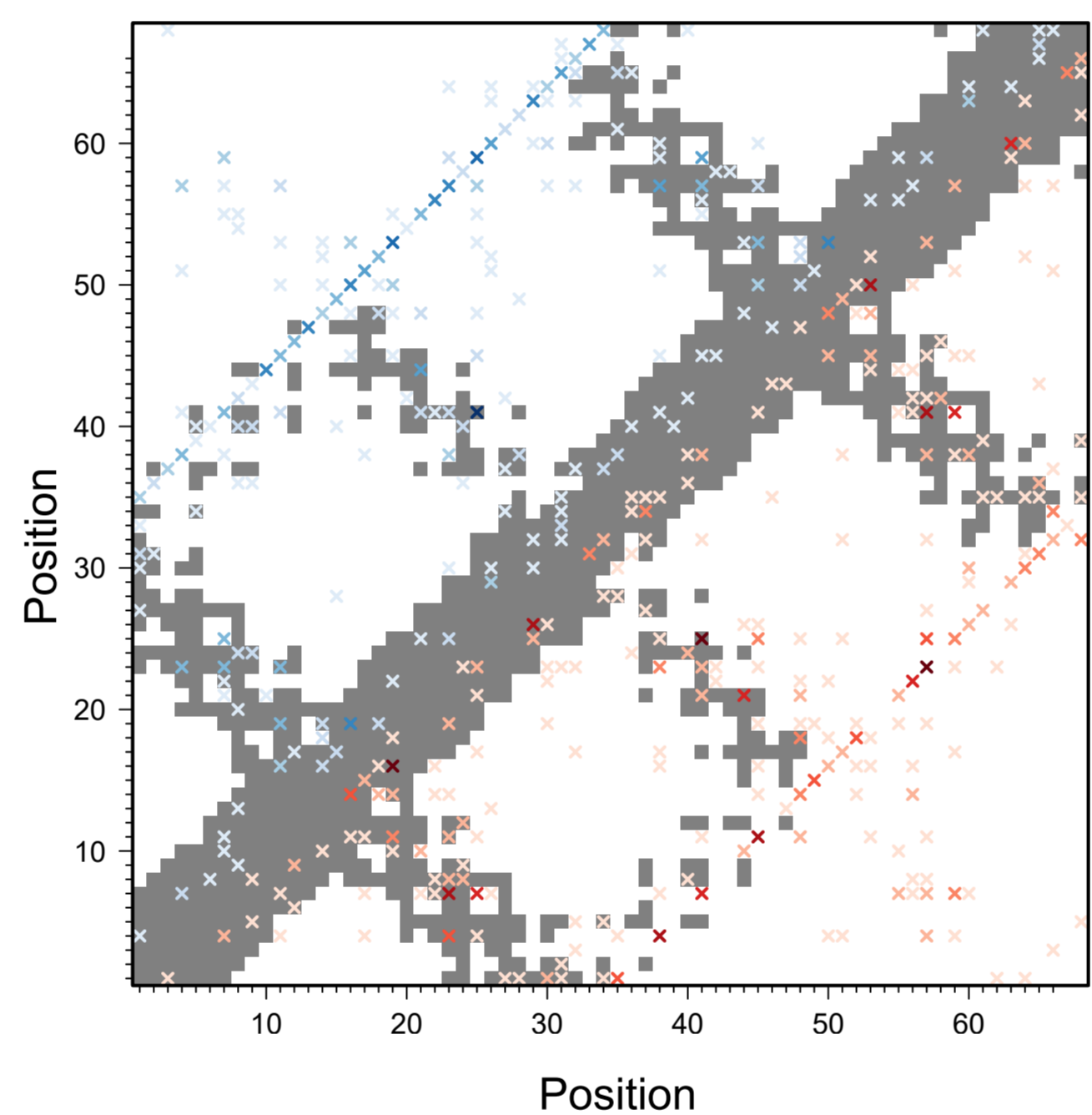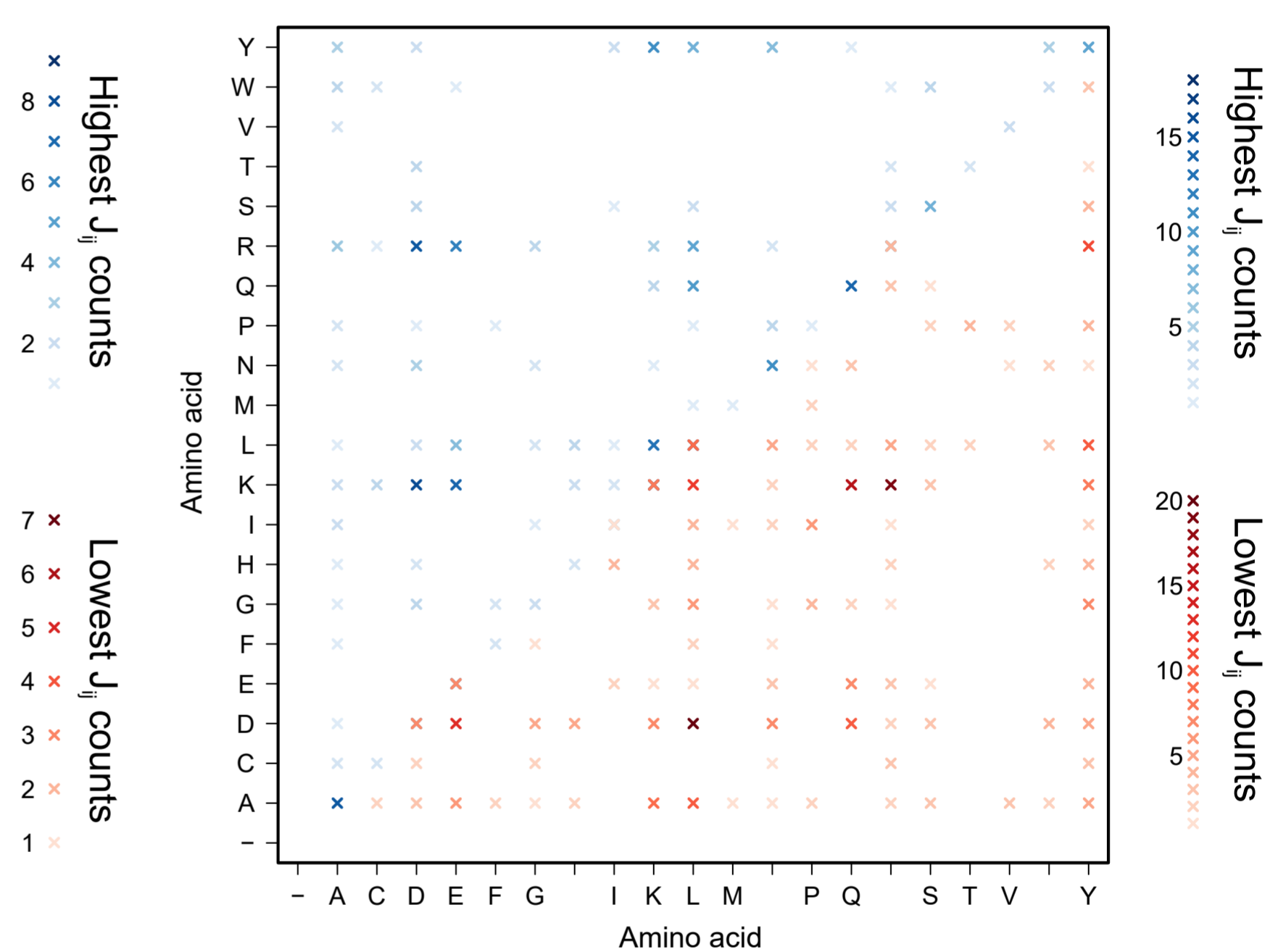

LRR

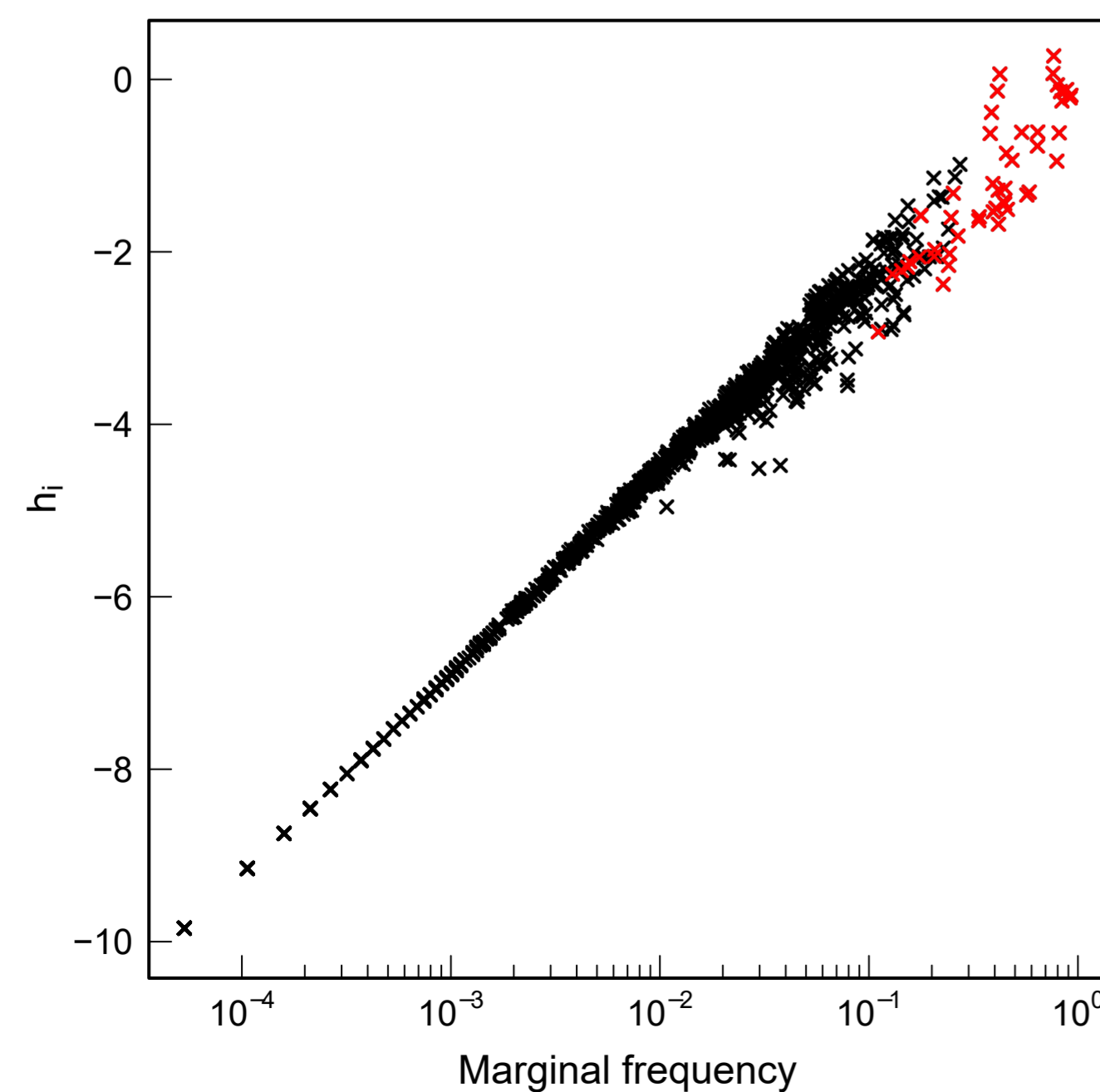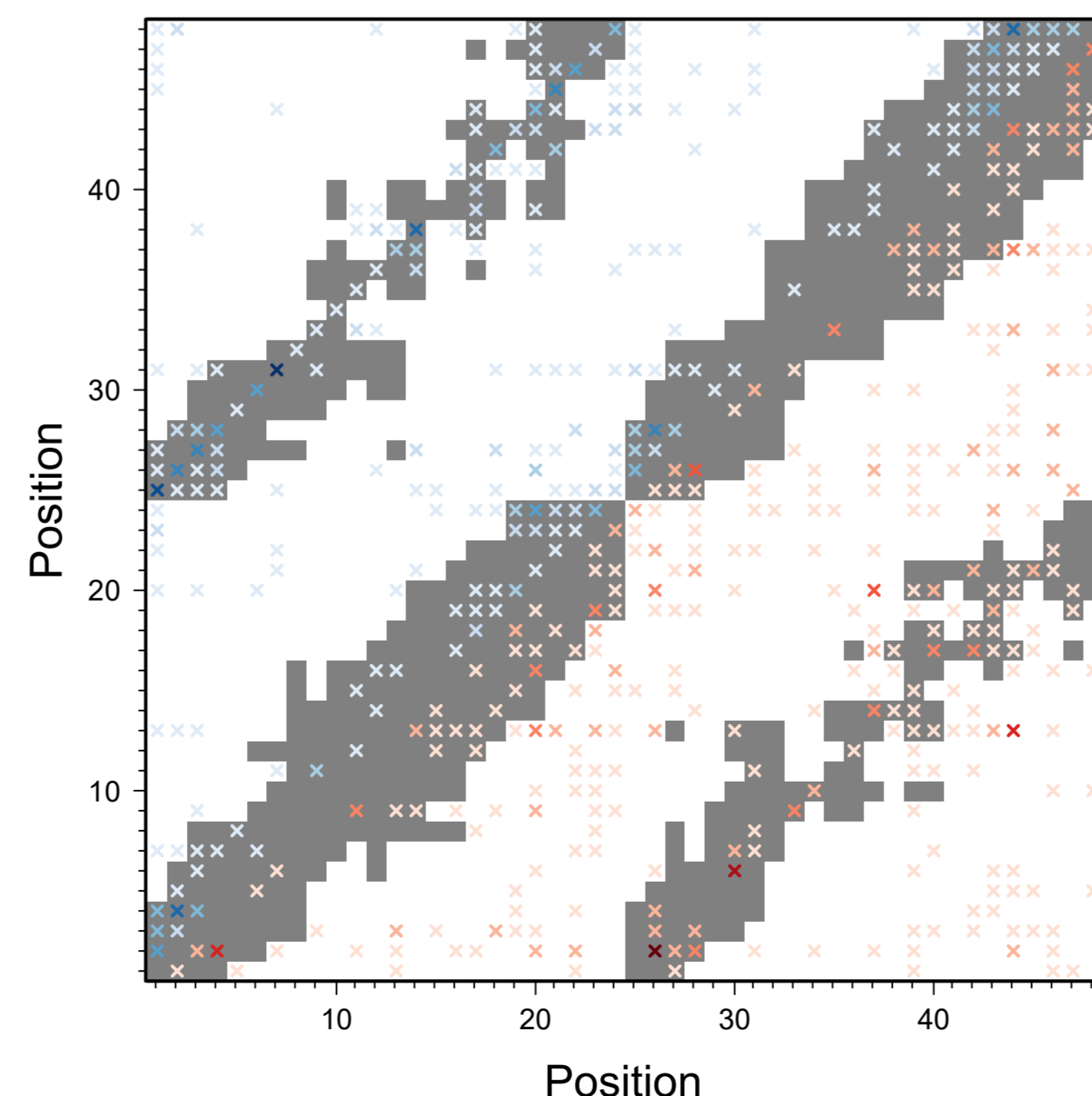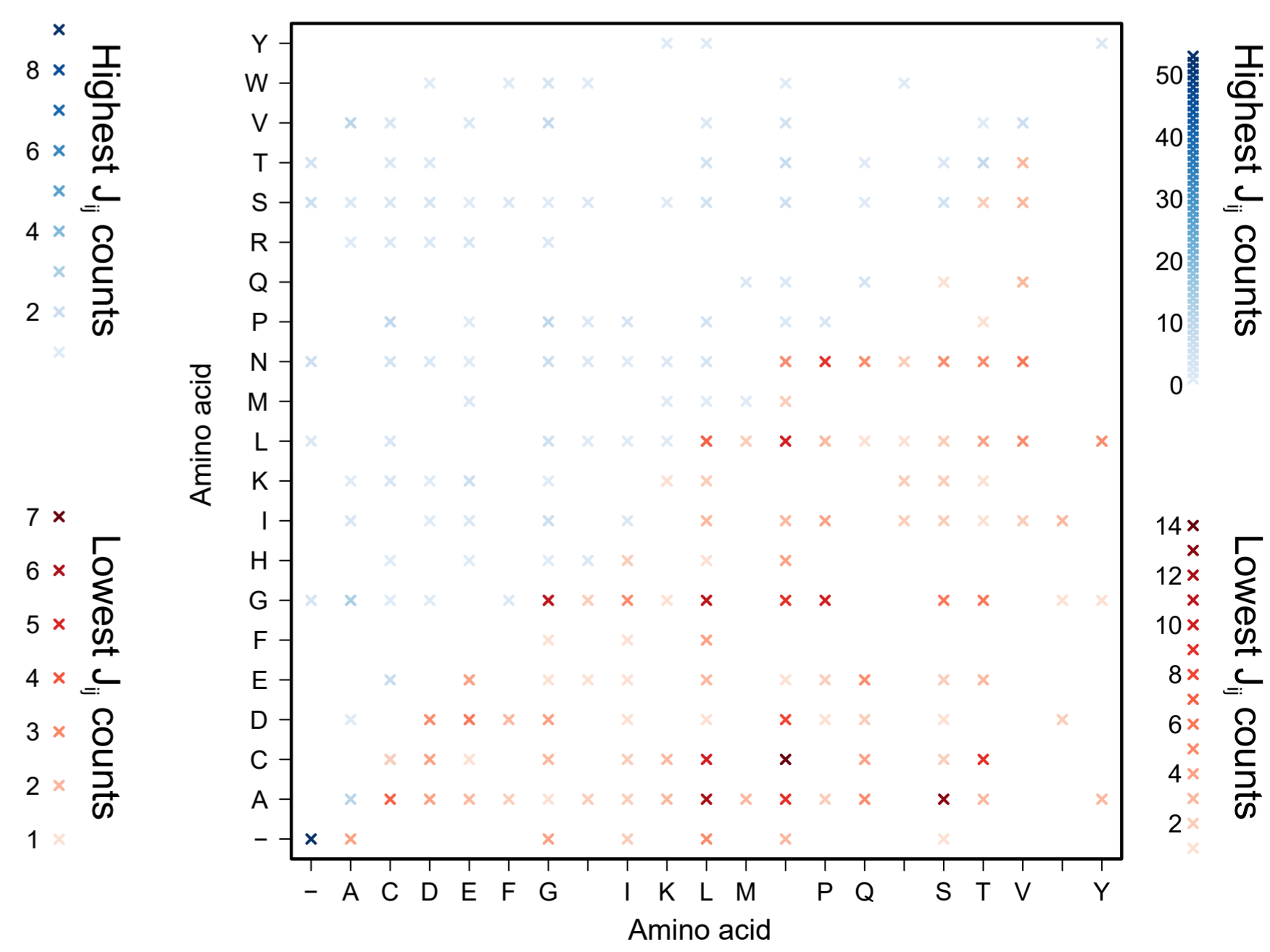

Supplement: S7 Fig — At left, comparison between the local field parameters hi(ai) and the marginal frequencies fi(ai). At center, contact map (grey indicates position in contact, white not in contact on the native structure). On blue, pairs of positions involved in highest Jij(ai, bj), red lowest Jij(ai, bj). At right, pairs of amino acids involved in the highest Jij(ai, bj) parameters (on blue) and in the lowest Jij(ai, bj) (on red). ANK at the top. TPR at the center. LRR at the bottom. (PDF) [file pcbi.1005584.s007.pdf]
